# AS1411 Aptamer Linked to DNA Nanostructures Diverts Its Traffic Inside Cancer Cells and Improves Its Therapeutic Efficacy

**DOI:** 10.3390/pharmaceutics13101671

**Published:** 2021-10-13

**Authors:** Giulia Vindigni, Sofia Raniolo, Federico Iacovelli, Valeria Unida, Carmine Stolfi, Alessandro Desideri, Silvia Biocca

**Affiliations:** 1 Department of Systems Medicine, University of Rome Tor Vergata, Via Montpellier 1, 00133 Rome, Italy; giulia.vindigni@uniroma2.it (G.V.); sofia.raniolo@uniroma2.it (S.R.); carmine.stolfi@uniroma2.it (C.S.); 2 Department of Biology, University of Rome Tor Vergata, Via della Ricerca Scientifica 1, 00133 Rome, Italy; federico.iacovelli@uniroma2.it (F.I.); valeria.unida@gmail.com (V.U.); desideri@uniroma2.it (A.D.)

**Keywords:** G-quadruplex, nucleolin, DNA nanocages, intracellular localization, cancer targeting, molecular dynamics simulations

## Abstract

The nucleolin-binding G-quadruplex AS1411 aptamer has been widely used for cancer therapy and diagnosis and linked to nanoparticles for its selective targeting activity. We applied a computational and experimental integrated approach to study the effect of engineering AS1411 aptamer on an octahedral truncated DNA nanocage to obtain a nanostructure able to combine selective cancer-targeting and anti-tumor activity. The nanocages functionalized with one aptamer molecule (Apt-NC) displayed high stability in serum, were rapidly and selectively internalized in cancer cells through an AS1411-dependent mechanism, and showed over 200-fold increase in anti-cancer activity when compared with the free aptamer. Comparison of Apt-NCs and free AS1411 intracellular distribution showed that they traffic differently inside cells: Apt-NCs distributed through the endo-lysosomal pathway and were never found in the nuclei, while the free AS1411 was mostly found in the perinuclear region and in nucleoli. Molecular dynamics simulations indicated that the aptamer, when linked to the nanocage, sampled a limited conformational space, more confined than in the free state, which is characterized by a large number of metastable conformations. A different intracellular trafficking of Apt-NCs compared with free aptamer and the confined aptamer conformations induced by the nanocage were likely correlated with the high cytotoxic enhancement, suggesting a structure–function relationship for the AS1411 aptamer activity.

## 1. Introduction

Aptamers are short single-stranded DNA or RNA oligonucleotides with stable three-dimensional structures capable of binding with high affinity to a variety of molecular targets [1]. A unique class of aptamers is constituted of G-rich sequences that, under physiological conditions, spontaneously fold into non-canonical four-stranded structures named G-quadruplexes. These are characterized by the stacking of two or more successive planes of four guanine residues arranged in a square planar array via Hoogsteen hydrogen bonding (a G-quartet). G-quadruplex structures are highly polymorphic, and a single sequence can fold into several different conformations, depending on the relative orientations of strands and types of loops [2,3,4]. The 26-base G-rich AS1411 aptamer has been identified as an anti-cancer agent, which specifically recognizes nucleolin on the cancer cell surface with high selectivity and affinity [5,6]. As other G-quadruplex aptamers, AS1411 adopts a mixture of multiple structures in solution, and one of them has been solved through NMR spectroscopy, studying a DNA sequence named AT11, where a single guanine has been substituted by a thymine. This mutated version shows a preferential G-quadruplex conformation and exhibits an anti-proliferative activity comparable to that of AS1411 [4,7].

The AS1411 target molecule, nucleolin, is a multifunctional cyto-nucleoplasmic protein mostly localized in the nucleoli in normal cells and aberrantly overexpressed in many types of cancers, where it is also located on the cell surface [8]. In cancer cells, the surface nucleolin acts as a receptor of oncogenic ligands and shows protumorigenic function regulating the biosynthesis of specific oncomiRNAs [9]. Following its interaction with nucleolin, AS1411 inhibits cancer cells proliferation and induces cell death, with little effects on normal cells. AS1411 exhibits antiproliferative effects by upregulating p53, downregulating Bcl-2, and inhibiting cancer cell migration and invasion in an Akt1-dependent manner [10,11,12]. Due to its unique properties, AS1411 has been used for its anticancer therapeutic effect and, when linked to nanoparticles, as a selective tumor-targeting ligand [13,14]. Notably, AS1411 aptamer treatment was evaluated in phase II clinical trials in patients with acute myeloid leukemia and renal cell carcinoma [15], but it never reached phase III.

In the last years, we have been involved in the in silico, in vitro, and in cell characterization of a DNA-based truncated octahedral nanocage family [16,17,18,19,20,21]. These nanocages are made by 12 double helices, obtained by assembling 8 oligonucleotides whose ends are covalently linked in order to produce fully covalent structures, which ensure high stability in biological fluids and inside cells. We have characterized the receptor-mediated cell internalization of DNA nanostructures and their efficacy in selective doxorubicin delivery and miRNA silencing in cancer cells [22,23,24]. When functionalized with folate molecules and tailored with anti-miR21 complementary sequences, DNA nanocages induce selective miR21 sequestering and cytotoxicity to tumor cells overexpressing the α isoform of the folate receptor [25].

In this work, with the aim of increasing AS1411 stability and therapeutic efficacy, we designed octahedral DNA nanocages harboring one AS1411 molecule (Apt-NC) and studied its efficiency as targeting ligand and cytotoxic molecule against cancer cells. We studied the in vitro and intracellular stability, the time-dependent cell internalization, the targeting selectivity, and the therapeutic efficacy of the new assembled nanocages. Interestingly, the AS1411-functionalized nanocages displayed higher cytotoxic efficacy and different intracellular trafficking than the free AS1411 aptamer. Molecular dynamics simulations of two 3D models of free AS1411 in comparison with the aptamer-functionalized nanocage indicated that the free aptamer sampled a large number of metastable conformations, while the conformations were more localized and confined into a low-energy conformational basin when linked to the nanocage. These results indicate that the nanocage constrains the aptamer in a more defined conformation, likely correlated to the different intracellular localization and higher therapeutic effect.

## 2. Materials and Methods

### 2.1. Preparation of Aptamer-Functionalized DNA Nanocages

AS1411-functionalized nanocages (Apt-NC) and non-functionalized nanocages (NC) were prepared as described [21]. A biotin molecule was added on one edge of the structure for the detection of nanostructures through the streptavidin–biotin reaction. Briefly, nanocages were assembled by mixing equimolar amounts of 8 oligonucleotides in TAM buffer (40 mM Tris–acetic acid, pH 7.0, 12.6 mM magnesium acetate). The sequences of the oligonucleotides used for the assembly of NCs and the sequence of free AS1411 aptamer are reported in Supplementary Table S1. After assembly, nanocages were incubated for 2 h at 25 °C with T4 DNA ligase (New England Biolabs Inc., Ipswich, MA, USA) to covalently link the obtained structures and run on native 5% polyacrylamide gels in TAEM buffer (40 mM Tris–acetic acid, pH 7.0, 1 mM EDTA, 12.6 mM magnesium acetate). The band corresponding to the correctly assembled nanocages was cut out of the gel, eluted, and concentrated by 2-propanol precipitation.

### 2.2. Cell Cultures

HeLa cells, derived from human cervix cancer, and CHO, Chinese hamster ovary, cells were grown in DMEM (Dulbecco’s modified Eagle medium, Euroclone, Devon, UK) and DMEM/F-12 (Dulbecco’s modified Eagle medium/Nutrient Mixture F-12, Euroclone, Devon, UK), respectively, supplemented with 10% FBS (Gibco, Paisleg, UK), 1 mM l-glutamine (Sigma Aldrich, St Louis, MO, USA), 1mM sodium pyruvate (Biowest, Miami, FL, USA), and 100 U/ml penicillin–streptomycin (Euroclone, Devon, UK).

### 2.3. DNA Nanocages Stability 

Biotinylated cages were incubated in TBS (Tris-HCl 50 mM, NaCl 150 mM, pH 7.8) or in culture medium supplemented with 10% FBS at 37 °C for different times. Each sample was then digested with proteinase K (100 μg/mL) for 1 h at 37 °C, and analyzed by DNA blot, as previously described [21]. Biotin detection was carried out using streptavidin–HRP (horseradish peroxidase) (Abcam Inc., Toronto, ON, Canada), and visualized by enhanced chemiluminescence (ECL Extend, Euroclone, Devon, UK). For image processing and densitometric analysis, photographic films were digitized by scanning and bands analyzed by ImageJ software.

### 2.4. Purification of DNA Nanocages and DNA Blot 

Cells were plated in 48-well plates one day before treatments. After incubation with DNA nanocages for different time periods, cells were lysed, centrifuged, digested with proteinase K, and DNA blot was performed as previously described [21].

### 2.5. Confocal Analysis

Cells were seeded onto poly-l-lysine-coated glass cover-slides. For binding experiments, cells were incubated with biotinylated nanocages in DMEM 10% FBS for 1 h at 4 °C, washed in PBS, fixed in 4% paraformaldehyde, and incubated for 5 min with NaBH_4_. For uptake experiments, cells were incubated with nanocages at 37 °C for different time periods, fixed with 4% paraformaldehyde, and permeabilized for 4 min with Tris/Triton (Tris HCl 0.1 M, Triton 0.1%, pH 7.7). Rabbit polyclonal anti-flotillin-1 antibody (Santa Cruz Biotechnology Inc., Dallas, TX, USA) was used to detect the flotillin-1 protein. Early endosomes were visualized with polyclonal EEA1 antibody (Abcam Inc., Toronto, ON, Canada) and lysosomes with mouse monoclonal anti-LAMP-1 antibody (Abcam Inc., Toronto, ON, Canada). Donkey anti-rabbit IgG and donkey anti-mouse IgG, both Rhodamine Red-X-conjugated AffiniPure (Jackson ImmunoResearch, Cambridgeshire, UK) were used as secondary antibodies [24]. Biotinylated cages were detected by using streptavidin–FITC (Jackson ImmunoResearch, Cambridgeshire, UK). The nuclei were stained with DAPI (Invitrogen, Carlsbad, CA, USA). Images were obtained with a laser confocal fluorescent microscope Olympus FV1000 at 60× magnification, and the fluorescence signal was evaluated with the IMARIS software. Co-localization events were evaluated as previously described [24].

### 2.6. Cell Viability Assay 

HeLa and CHO cells were plated in 96-well plates at a density of 5 × 10^3^ cells/well and 7 × 10^3^ cells/well, respectively, grown for 24 h, and treated with different concentrations of Apt-NCs, NCs, or free AS1411 aptamer for 24 h at 37 °C. The MTS assay (3-(4,5-dimethylthiazol-2-yl)-5-(3-carboxymethoxyphenyl)-2-(4-sulfophenyl)-2H-tetrazolium) (Pro-mega, WI, USA) was used for evaluating cell proliferation. Multiskan Ascent 96/384 plate reader (MTX Lab Systems, Bradenton, FL, USA) was used for measuring the absorbance at 492 nm. Cell viability was normalized to control condition (untreated cells).

### 2.7. Statistical Analysis

All experiments were carried out in triplicate and data analyzed using GraphPad Prism. Results were expressed as a mean ± S.E.M and statistical analyses performed using Student’s *t*-test. Differences were considered statistically significant when *p* < 0.05 (*), *p* < 0.01 (**) and *p* < 0.001 (***).

### 2.8. Computational Methods

Two models of the AS1411 G-quadruplex were generated. The first one (Supplementary Figure S1, AS1411) was generated starting from the PDB structure with ID:2N3M corresponding to the AT11 variant of the aptamer [4] using the fiber module of the 3DNA program [26] to generate the PDB file. The nucleotide sequence of the strand composing the G-quadruplex was modified through the 3DNA mutate_bases module [26] to fully match the AS1411 oligonucleotide sequence. The PyMol sculpting module [27] was used to generate an additional conformation, imposing guanines 11 as part of the first G-quartet of the aptamer (Supplementary Figure S1, AS1411*). The structures were minimized using the UCSF Chimera program [28] to remove any clashes and unwanted interactions introduced by the modeling procedure. The octahedral scaffold of the DNA nanocages was built through our Polygen software [16] designing eight oligonucleotide sequences (Supplementary Table S1) based on those previously used to experimentally assemble different truncated octahedral geometries [22,29]. The AS1411-NC and AS1411*-NC structures were modeled using the SYBYL 6.0 program (TRIPOS, http://www.tripos.com, accessed on 30 June 2021), manually adding one copy of the AS1411 aptamer to the octahedral scaffold, using a 7-thymidine spacer to keep the structure accessible for nucleolin targeting. The system topologies and the coordinates of AS1411, AS1411*, AS1411-NC, and AS1411*-NC models, used as input for the AMBER 16 MD package [30], were generated through the AmberTools tLeap module, parametrizing the structures through the AMBER ff19SB force field with the parmbsc1 corrections [31]. The structures were immersed in a rectangular box filled with TIP3P water molecules, imposing a minimum distance between the solute and the box of 14 Å, neutralizing the charges adding Mg^2+^ counterions to the solvated systems in favorable positions, as implemented in the tLeap program, together with 4 K^+^ ions for G-quadruplex stabilization [32]. All systems were minimized using 2500 steps of the steepest descent algorithm to remove unfavorable interactions and prevent Mg^2+^ ions from binding to DNA, applying harmonic restraints of 50 kcal·mol^−1^·Å^−2^. The systems were then equilibrated to 300 K in the NVT ensemble for 500 ps using the Langevin thermostat, with a coupling coefficient of 1.0 ps and including cartesian restraint of 15 kcal·mol^−1^·Å^−2^ on nucleotide atoms. After the equilibration phase, the systems were subjected to an equilibrium simulation of 500 ps removing all constraints and to relax all atoms. The systems were then simulated using the NPT ensemble for a period of 10 ns, applying periodic boundary conditions, a 2.0 fs time-step, and the PME method [33] for the long-range electrostatic interactions with a cutoff of 9.0 Å for the evaluation of short-range nonbonded interactions. The SHAKE algorithm [34] was used to constrain covalent bonds involving hydrogen atoms. The temperature was fixed at 303 K using the Langevin dynamics [35], while pressure was kept constant at 1 atm through the Langevin piston method [36]. Atomic positions were saved every 1000 steps (2.0 ps) for the analyses.

### 2.9. Gaussian Accelerated Molecular Dynamics Simulations

For each system, 100 ns dual-boost Gaussian accelerated molecular dynamics (GaMD) simulations [37] were performed, saving the atomic positions every 1000 steps. GaMD is an enhanced sampling method in which a harmonic boost potential is applied to smooth the potential energy surfaces and reduce the systems energy barriers [37]. Using this approach, it is possible to sample a large conformational space, which cannot be normally accessed by classical MD simulations. Moreover, by constructing a boost potential following Gaussian distribution, using cumulant expansion to the second order, GaMD simulations can be correctly reweighted to recover the original biomolecular free energy profiles [37]. GaMD simulations were entirely performed using an NVIDIA TITAN XP GPU using the default parameters suggested for the method (http://miao.compbio.ku.edu/GaMD/, last accessed 30 June 2021).

### 2.10. Trajectory Analysis 

The GROMACS 2020.3 analysis tools [38] were used to compute the root mean square fluctuations (RMSFs) and principal component analysis (PCA) over the entire 100 ns trajectories. A clustering analysis was performed on all the saved configurations through the gmx cluster module of GROMACS using the GROMOS algorithm [39], forcing a cut-off of 0.12 nm for the geometrical clustering procedure. In order to recover the original free-energy profiles of the simulated structures, the GaMD trajectories were reweighted using PyReweighting, a toolkit of Python scripts designed to facilitate the GaMD simulation analyses [37]. The UCSF Chimera program [28] was used to generate the pictures of the representative structures.

## 3. Results

### 3.1. Design, Assembly, and In Vitro Stability of AS1411 Functionalized DNA Nanocages

**Figure 1:**
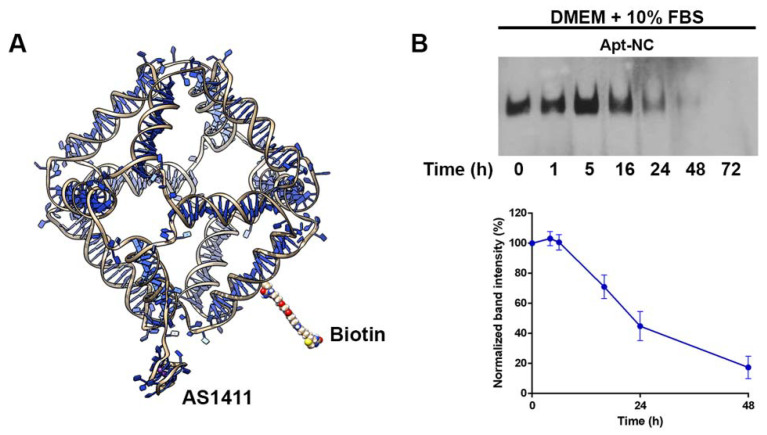
(**A**) Schematic representation of the Apt-NC nanostructure model. (**B**) DNA blot analysis of Apt-NCs incubated in DMEM supplemented with 10% FBS at 37 °C for different times, as indicated. Apt-NCs before incubation with serum proteins are indicated as time 0. Biotinylated Apt-NCs were detected with streptavidin–HRP. The graph shows the densitometric analysis of three different experiments, performed using the ImageJ software. The relative intensity of each band was normalized to the intensity of the band corresponding to time 0 and reported in the graph. Values are expressed as an average ± SEM.

AS1411 has been shown to adopt multiple conformations characterized by very similar hydrodynamic and electrophoretic properties [7]. A single G-quadruplex conformation, named AT11, can be obtained by mutating a guanine in thymine in the position 10 [4]. The AT11 structure was obtained by NMR spectroscopy, and we started from this PDB structure to build the AS1411 atomic 3D model, introducing the original AS1411 sequence through the 3DNA mutate_bases module (Supplementary Figure S1) [26]. A second 3D model of the aptamer, called AS1411*, was generated replacing guanine 10 with guanine 11 in the formation of the first G-quartet of the aptamer (Supplementary Figure S1). A 3D model with one AS1411 or AS1411* aptamer (Apt) attached to a DNA nanocage (NC) was built to form the Apt-NC structure (Figure 1A). The octahedral nanocage was obtained from 8 oligonucleotide sequences (OL1–OL8, Supplementary Table S1) to form a scaffold made up of 12 double stranded B-DNA helices forming the edges of a covalently closed octahedron, connected by short single-stranded five-thymidine linkers, corresponding to the square truncated faces [21,23,25]. AS1411 aptamer was connected to the nanocage scaffold through a seven-nucleotide linker made by thymidine, using a modified oligonucleotide (OL8_Apt_) (Supplementary Table S1).

The assembly of AS1411-octahedral nanocages was performed as described [21], using the oligonucleotides reported in Supplementary Table S1. In electrophoresis, the assembled nanocages ran in the gel as a single well-defined product with a molecular weight of about 530 bp (Supplementary Figure S2). As depicted in Figure 1A, Apt-NCs were functionalized with a biotin molecule on one edge of the structure. The presence of biotin enabled detection of the assembled nanocages after transfer from the gel to a membrane, through the streptavidin (HRP)–biotin reaction in the DNA blot, a system with a sensitivity up to 100 times higher than the ethidium bromide staining, as we calculated in a comparative test.

Figure 1B shows the stability of biotinylated Apt-NCs incubated at 37 °C in DMEM cell culture medium supplemented with 10% FBS for different time points. After incubation, each sample was treated with proteinase K (100 μg/mL), run in 5% polyacrylamide gel, and analyzed in DNA blot using streptavidin–HRP [21]. Apt-NCs were stable and fully intact for at least 5 h of incubation with serum, then they started to be slowly degraded as a function of time and were still detectable after 48 h (Figure 1B, top panel). In detail, the half-life of Apt-NCs in DMEM–10% FBS was 25 h, calculated by the relative intensity of each band visualized through DNA blot (Figure 1B, bottom panel). Since K^+^ ions act as stabilizing agents for G-quadruplex structures [40], we tested the in vitro stability of Apt-NCs in serum in the absence or in the presence of 5 mM KCl. DNA blot analysis showed that the presence of only K^+^ ions increased the resistance of Apt-NCs to serum nucleases, extending their half-life from 16 to 22 h (Supplementary Figure S3). Notably, the presence of both monovalent and bivalent cations in DMEM culture medium guaranteed excellent stability to Apt-NCs (Figure 1B).

### 3.2. Intracellular Stability and Targeting Efficiency

**Figure 2:**
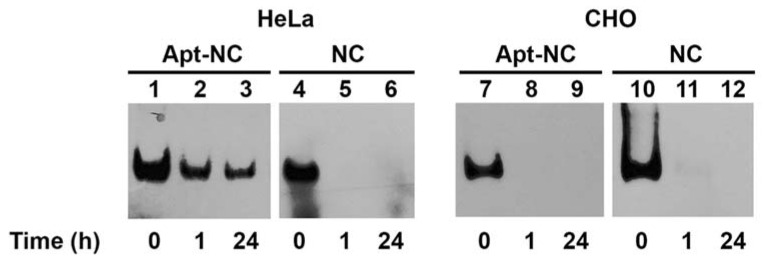
DNA blot of Apt-NC (lanes 2–3 and 8–9) and non-functionalized NC (lanes 5–6 and 11–12) purified from HeLa and CHO cell extracts after 1 and 24 h incubation. In lanes 1, 4, 7, and 10, 30 ng of nanocages before incubation with cells (time 0) are shown. Biotinylated nanostructures were detected with the streptavidin (HRP)–biotin reaction.

The targeting efficiency and intracellular stability of AS1411-modified NCs were studied by DNA blot in HeLa cells, a human cervical cancer cell line with high surface nucleolin expression [41]. Figure 2 shows HeLa cells incubated with 30 nM biotinylated Apt-NCs or non-functionalized NCs (NC) for 1 and 24 h at 37 °C. After incubation, nanostructures were purified from cell lysates and analyzed by DNA blot, using streptavidin–HRP [21]. Lanes 1, 4, 7, and 10 show the band corresponding to DNA nanocages before incubation with cells (time 0). After purification from cell extracts, Apt-NCs ran as one band in the gel, with a mobility comparable to the input (time 0), confirming that we were visualizing only Apt-NCs that were intact inside cells. Interestingly, a similar band intensity was detected at 1 and 24 h of incubation (compare lanes 2–3), suggesting that Apt-NCs were rapidly internalized inside cells, but they did not accumulate in a time-dependent manner. Notably, NCs without aptamer functionalization were almost non-detectable (lanes 5–6), demonstrating that efficient cell uptake occurred only through a nucleolin-mediated AS1411-dependent mechanism. As a control, non-cancer CHO cells, which express very low amount of membrane nucleolin [42], did not show any significant entry of nanocages after 1 and 24 h of incubation with either biotinylated Apt-NCs (lanes 8–9) or non-functionalized NCs (lanes 11–12).

### 3.3. Intracellular Trafficking of Apt-NCs 

**Figure 3:**
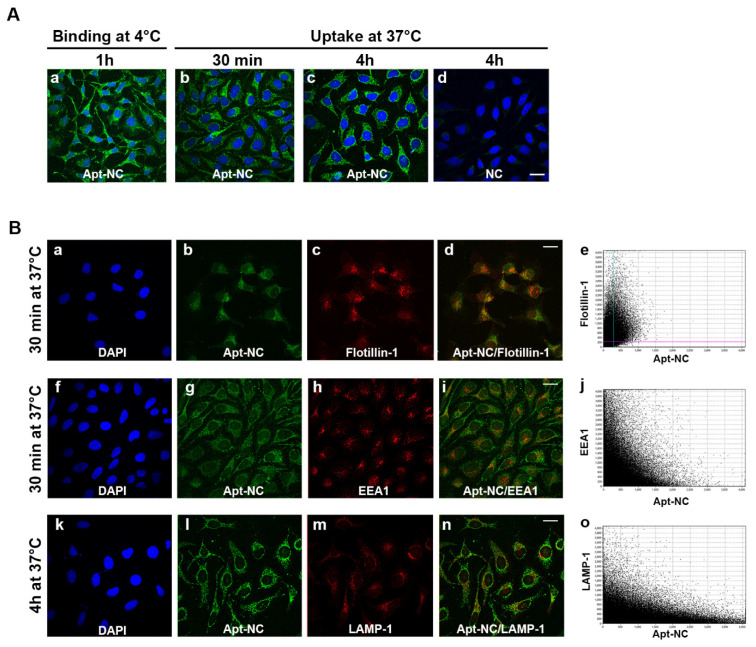
(**A**) Confocal analysis of membrane-binding and intracellular distribution of Apt-NCs. HeLa cells were incubated with 60 nM of biotinylated Apt-NCs (panels **a**–**c**) or non-functionalized NCs (panel **d**) for 1 h at 4 °C (panel **a**) or for 30 min and 4 h at 37 °C (panels **b**–**d**), as indicated. (**B**) Co-localization analysis of Apt-NCs (panels **b**,**g**,**l**) with flotillin-1 (panel **c**), endosomal marker EEA1 (panel **h**), and lysosomal marker LAMP-1 (panel **m**). The (**b**,**c**) images are merged in (panel **d**,**g**,**h**) images are merged in (panel **i**), and the (**l**) and m images are merged in (panel **n**). Cell nuclei are visualized in (panels **a**,**f**,**k**). The correlation of the intensity values of the green and red pixels was performed using the IMARIS software and scatter plots are reported in (panels **e**,**j**,**o**). Biotinylated NCs were visualized using streptavidin–FITC and nuclei were stained with DAPI. Scale bar: 20 μm.

The membrane binding and cellular internalization of AS1411-functionalized NCs were studied by confocal microscopy in HeLa cells. To distinguish between membrane-bound and internalized nanostructures, cells were incubated at 4 °C or at 37 °C, respectively. At 4 °C, nanostructures can only enter the cell through direct translocation, because endocytosis, an energy-dependent process, is inhibited at this temperature. Moreover, for visualizing the membrane-bound nanostructures, cells were only fixed, whilst for studying the internalized NCs, cells were incubated for different time periods, then fixed and permeabilized. Representative confocal images of biotinylated-Apt-NC binding at 4 °C and uptake at 37 °C are shown in Figure 3A. Apt-NCs efficiently bound to HeLa cells and appeared as many small green fluorescent dots on the plasma membrane (Figure 3A, panel a). At 37 °C, after 30 minutes, most of the green fluorescent dots were already visible inside cells (Figure 3A, panel b) and remain visible after 4 h of incubation (Figure 3A, panel c), showing a vesicular distribution. In line with DNA blot experiments shown in Figure 2, very low fluorescent signal is observed in HeLa cells treated with NCs (Figure 3A, panel d), confirming that nanocages are entering in cells through a AS1411-dependent mechanism.

The nucleolin-dependent internalization mechanism was clarified by performing a double fluorescence staining of Apt-NCs and Flotillin-1, a lipid raft marker protein which associates with nucleolin in membrane lipid rafts [43]. As shown in Figure 3B (panels a–e), after 30 min of incubation, the green punctate staining of Apt-NCs (panel b) significantly overlapped with the red fluorescence of flotillin-1 (panel c), as confirmed by merged images of green and red fluorescence in panel d. The correlation of the intensity values of the green and red pixels is reported in the scatter plot (panel e) used to calculate the Pearson’s coefficient (PCC) [44]. The PCC was 0.47 ± 0.03, confirming that Apt-NCs were internalized through a nucleolin-mediated mechanism involving flotillin endocytosis.

The intracellular distribution was further studied by colocalization analysis of Apt-NCs with the early endosomal antigen (EEA1) after 1 h incubation or the lysosomal-associated membrane protein 1 (LAMP-1) [24] after 4 h incubation. Figure 3B shows representative images of double immunofluorescence of Apt-NCs, shown as green dots inside cells (panels g and l), and EEA1 and LAMP-1 (panels h and m) as red dots. The intracellular distribution was only partially overlapping, as visualized in merged images (panels i and n) and the corresponding scatter plots (panels j and o). The correlation of the intensity values of the green and red pixels evaluated by the Pearson’s coefficient (PCC) [44], analyzing the scatter plot, is shown in panels e and j. In detail, the PCC was 0.20 ± 0.02 with the EEA1 marker after 30 min of internalization and 0.27 ± 0.01 with the LAMP-1 marker after 4 h, indicating that Apt-NCs partially trafficked through the endo-lysosomal pathway.

**Figure 4:**
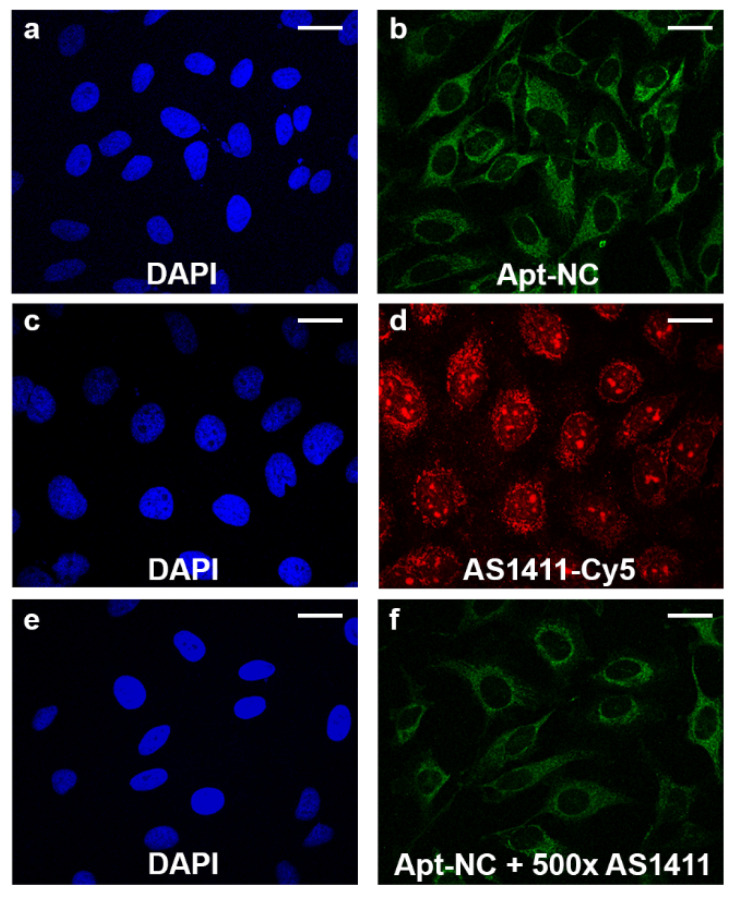
Comparison of intracellular distribution of Apt-NCs and Cy5-AS1411. Cells were incubated with 60 nM biotinylated Apt-NCs (panels **a**,**b**) or 3 µM free Cy5-AS1411 (panels **c**,**d**) for 1 h at 37 °C. (panels **e**,**f**) show cells incubated with Apt-NCs in the presence of 500 molar excess of free AS1411. Biotinylated Apt-NCs were visualized using streptavidin–FITC, and nuclei were stained with DAPI. Scale bar: 20 μm.

Guided by these observations, we compared the intracellular distribution of free AS1411 and Apt-NC in HeLa cells and verified whether the presence of a large excess of free AS1411 was able to impair the uptake and the intracellular distribution of Apt-NCs. HeLa cells were incubated with Apt-NCs (Figure 4, panels a and b) or AS1411-Cy5 (Figure 4, panels c and d) for 1 h at 37 °C and analyzed by confocal microscopy. Apt-NCs appeared as green fluorescent punctate staining in the cytoplasm (Figure 4, panel b), whilst free AS1411-Cy5 appeared as red dots mostly localized in nucleoli and in the perinuclear region (Figure 4, panel d). It was evident that Apt-NCs and free AS1411 aptamer trafficked inside cells differently and that the AS1411-functionalized nanocages were never found in the nuclei.

Interestingly, incubation of Apt-NCs in the presence of 500 times molar excess of free AS1411 (Figure 4, panel f) did not lead to significant reduction of the green signal (compare panels b and f), as also confirmed by green fluorescence intensity profiles of the confocal images (Supplementary Figure S4), indicating that cell uptake of Apt-NCs was not impaired by the presence of a large excess of free aptamer and demonstrating that the receptor-mediated uptake of AS1411-linked to the nanocages in HeLa cells had a higher efficiency compared with the free aptamer. All together, these results suggest that AS1411-NC functionalization may facilitate AS1411-nucleolin or other membrane proteins interactions, activating a different entry pathway.

### 3.4. Cytotoxic Effect of Apt-NCs

**Figure 5:**
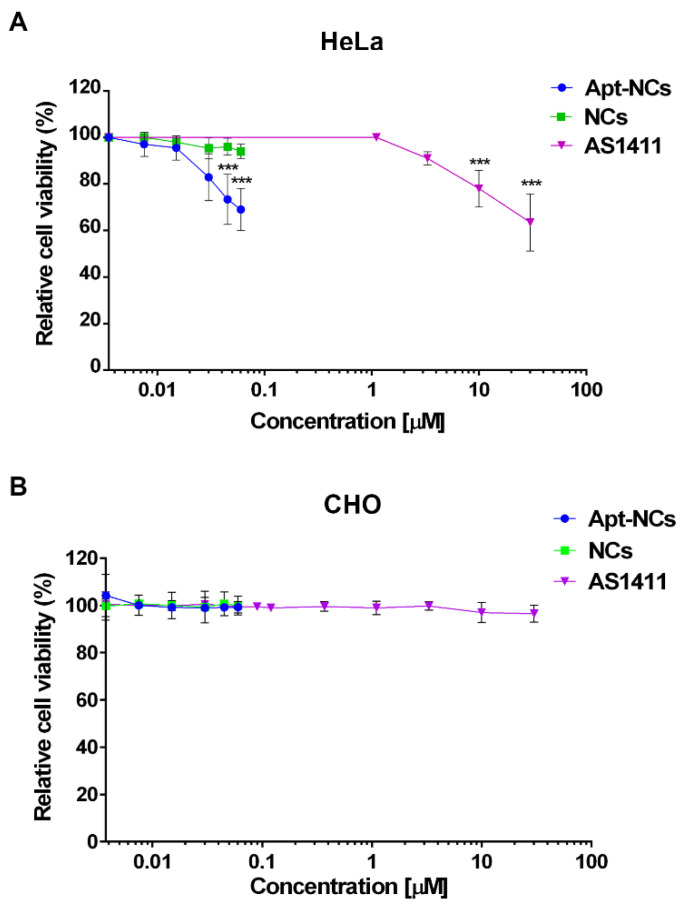
Cytotoxic effect of Apt-NCs in HeLa (**A**) and CHO cells (**B**). Cell proliferation after 24 h treatment with Apt-NCs, non-functionalized NCs, and free AS1411 at different concentrations was assessed by MTS. The values are the means of six replicates normalized to untreated cells. Statistical significance: (***) *p* < 0.001 (Student’s *t*-test) compared with NCs treated cells in the case of Apt-NCs and untreated cells in the case of AS1411.

The cytotoxic activity of Apt-NCs was tested in comparison with non-functionalized NCs and free AS1411 in cancer HeLa and non-cancer CHO cells. Cells were treated for 24 h with NCs at concentrations ranging from 3.75 nM to 60 nM and with free AS1411 at concentrations ranging from 3.75 nM to 30 µM (Figure 5). Figure 5A shows that incubation of HeLa cells with free AS1411 aptamer was not cytotoxic up to micromolar concentrations. On the other hand, we observed a dose-dependent reduction of cell viability of HeLa cells when treated with Apt-NCs already in the nanomolar range, indicating more than 200-fold increase in cytotoxicity, in comparison with the free aptamer (Figure 5A). No significant reduction in cell viability was observed when treating HeLa cells with NCs not functionalized with the aptamer. As a control, we analyzed the cytotoxic effect of Apt-NCs and free AS1411 on the non-cancer cell line CHO (Figure 5B). No reduction in cell proliferation was observed in CHO cells at all tested concentrations, demonstrating that AS1411 linked to DNA nanocages is not toxic in non-cancer cells. In agreement with this result, free AS1411 induced low cytotoxicity in CHO as in other non-cancer cells [42]. Flow cytofluorimetry was performed for detecting apoptotic cells by a double staining with annexin V–FITC/propidium iodide (PI) (Supplementary Figure S5). In detail, after 24 h treatment with Apt-NCs the percentage of apoptotic cells reached 33.2 ± 1.2% at 45 nM, while in cells treated with pristine NCs at the same concentration, the percentage was 9.8 ± 1.9%, similar to that observed in untreated control cells (9.9 ± 0.3%). The analysis confirmed the MTS results, showing that Apt-NCs induced a significant dose-dependent apoptosis in HeLa cells while no effect was observed in non-cancer CHO cells.

### 3.5. GaMD Simulations

**Figure 6:**
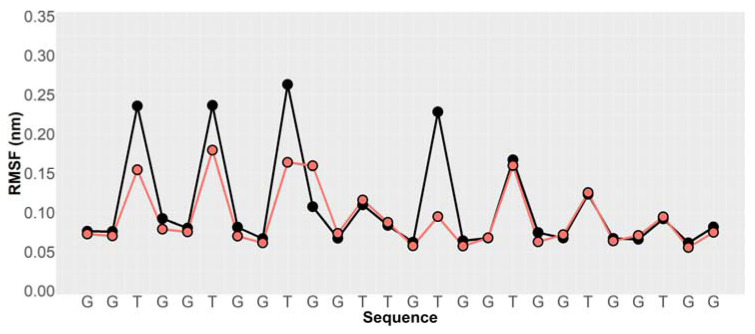
RMSF values calculated for the C2′ atoms of the AS1411 aptamer. Black and red filled circles indicate the values calculated for the free and cage-linked aptamers, respectively.

**Figure 7:**
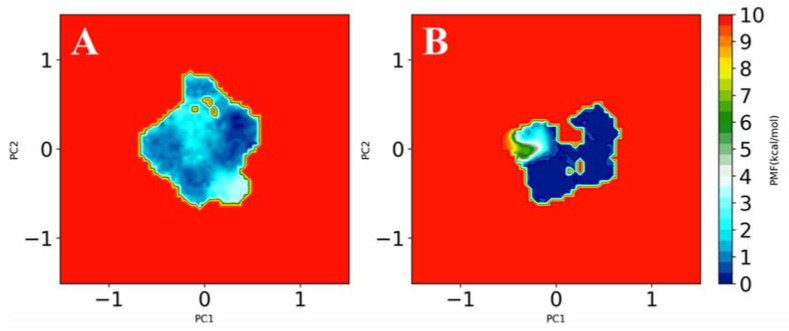
Free energy principal component projection of the free (**A**) and cage-linked (**B**) AS1411 aptamers.

G-quadruplex-forming sequences are remarkably polymorphic. A single sequence may reach a different 3D structure depending on the physicochemical conditions, such as different folding conditions or presence of a different counterion bound in its core. The high-resolution 3D structure of AS1411 has never been reported due to the high complexity resulting from the simultaneous presence of several conformers that has been demonstrated through multiple spectroscopic techniques [7]. We performed four 100 ns long GaMD simulations, two for each model generated for the free aptamers (AS1411 and AS1411*) and two for the models of the aptamers linked to the octahedral nanocage (AS1411-NC and AS1411*-NC), in the presence of potassium ions. Figure 6 shows that the RMSF values, describing the time-averaged deviation of C2′ atom positions of the nucleotides, were higher for the free AS1411 (black line) than for the NC-linked aptamer (AS1411-NC) (red line), suggesting a lower conformational space sampling for the latter structure. A similar result was obtained comparing the RMSF of the free AS1411* and of the AS1411*-NC models (Supplementary Figure S6). A PCA analysis of the motions, coupling the projection of the first two main motions to the reweighting of the GaMD simulations, was carried out to recover the original free energy profile of the molecules (Figure 7 and Supplementary Figure S6). In this representation, the conformational space sampled by the structure is directly proportional to the number of points plotted on the graph, while the color indicates the energy of the sampled conformations, whose values increase from blue to red. The free AS1411 and AS1411* structures (Figure 7A and Supplementary Figure S7A) sampled several metastable conformations, characterized by small energy differences of about 1–2 kcal/mol. On the other hand, in the AS1411-NC and AS1411*-NC structures, the aptamer sampled a more localized conformational space, confined into low-energy basins (Figure 7B and Supplementary Figure S7B). Indeed, by clustering the different frames obtained from the four trajectories, we found two main representative clusters for the AS1411-NC and AS1411*-NC (Supplementary Figure S8, green and yellow bars), while the two free aptamer structures were characterized by more than 40 low-populated different clusters (Supplementary Figure S8, red and blue bars), confirming a much lower conformational variability for the aptamer linked to the nanocage scaffold.

## 4. Discussion

Here we report a marked enhancement of the G-quadruplex AS1411 therapeutic efficiency due to its functionalization over octahedral DNA nanocages. Aptamer-linked nanocages (i) are stable both in serum and inside cells, similarly to other previously characterized covalently linked DNA nanostructures [21,22,29], (ii) are selectively and efficiently internalized by cancer cells, and (iii) increase the cytotoxic efficacy by more than two orders of magnitude compared with a free aptamer. At least two main explanations can be conceived for the enhanced AS1411 cytotoxicity observed in cancer cells when linked to DNA nanostructures: (i) a more defined conformation adopted by the cage-linked AS1411 and (ii) a different intracellular entry pathway in cancer cells followed by the cage-linked AS1411 compared with free aptamer.

Analysis of the conformational variability of two 3D models of the free aptamers (AS1411 and AS1411*) and of the aptamers linked to the octahedral nanocage (AS1411-NC and AS1411*-NC) through GaMD simulations showed that the free AS1411 or AS1411* aptamers generated more than 40 conformational clusters, indicating that the free aptamer samples many metastable conformations, characterized by small energy differences. On the other hand, MD simulations of Apt-NC pointed out that the nanocage scaffold constrains the aptamer to adopt a more defined conformation, independently of the starting structure, mainly described by two representative clusters (Supplementary Figure S6). Consequently, the structure of the free aptamer appears to be more floating, as confirmed by the RMSF analyses. These results are likely correlated to the higher efficiency of the cage linked AS1411 in targeting nucleolin and/or other membrane proteins in cancer cells and in improving cell toxicity compared with the free aptamer. Indeed, this may represent an example of structure–function relationship, since the enhanced AS1411 cytotoxicity appears correlated to a confinement of the aptamer in a more defined conformation than that of the free aptamer. 

AS1411 has been linked to various types of nanoparticles due to its selective binding to nucleolin, a protein overexpressed on the cell surface and inside cancer cells [45,46,47,48,49]. Notably, a marked improvement of cytotoxicity has been previously obtained by increasing the loading (more than 100 molecules) of AS1411 on the surface of gold nanostars [50]. However, the here described inhibition of cancer cell proliferation by AS1411 linked to NCs (up to two orders of magnitude greater than that by the free aptamer) is the most powerful reported so far.

We demonstrated that the aptamer AS1411 confers a selective targeting activity to Apt-NCs, when compared with pristine nanocages, showing a clear targeting preference for the nucleolin-positive HeLa cancer cells compared with the nucleolin-negative control cells. Apt-NCs are efficiently taken up by cancer cells using a flotillin-dependent endocytosis mechanism, traffic through the endo-lysosomal pathway, and never reach the nuclei, whilst AS1411 traffics both to the cytoplasm and to the nuclei, accumulating in nucleoli. By competition experiments, we also demonstrated that aptamer-linked nanocages display a higher internalization efficiency than free AS1411. The different intracellular traffic and localization may be responsible for the higher cytotoxicity, changing the mechanism of action of AS1411, although further studies are required to definitely confirm it and to identify the pathway.

The proposed AS1411-functionalized DNA nanocages are very stable and, beyond the marked increase of cytotoxicity to cancer cells, present the advantage of being a potential multifunctional scaffold. In fact, the octahedral DNA nanocages can be tailored with sequestering units for selective oncomiR inhibition [25] and/or loaded with DNA intercalating drugs, such as doxorubicin [23,25], and used as multipurpose delivery vehicles.

## Data Availability

Not applicable.

## Supplementary Materials

The following are available online at https://www.mdpi.com/article/10.3390/pharmaceutics13101671/s1, Table S1: Sequences of the oligos for the assembly of octahedral DNA nanocages, Figure S1: Schematic representation of G-quadruplex folding topology of AS1411 and AS1411* in K^+^ solution, Figure S2: Gel electrophoresis analysis of pristine (NCs) and AS1411-functionalized nanocages (Apt-NCs), Figure S3: Stability of Apt-NCs in the absence or presence of K^+^ ions, Figure S4: Intensity profiles of streptavidin–FITC signal, Figure S5: Annexin V-FITC/propidium iodide assay in HeLa and CHO cells, Figure S6: RMSF values calculated for the C2′ atoms of the AS1411* structures, Figure S7: Free energy principal component projection of the isolated (A) and cage-linked (B) AS1411* aptamers, Figure S8: Bar chart representing the distribution of the clustered AS1411, AS1411-NC, AS1411*, and AS1411*-NC conformations.
