# Supplementary material for: AS1411 Aptamer Linked to DNA Nanostructures Diverts Its Traffic Inside Cancer Cells and Improves Its Therapeutic Efficacy
Source: Pharmaceutics. 2021 Oct 13;13(10):1671. doi: 10.3390/pharmaceutics13101671 (PMC8541364; doi:10.3390/pharmaceutics13101671)
Supplement: Supplementary file 1 [file pharmaceutics-13-01671-s001.zip › pharmaceutics-1393712-supplementary.pdf]

pharmaceutics

MDPI

Supplementary Materials: As1411 Aptamer Linked to DNA Nanostructures Diverts Its Traffic Inside Cancer Cells and Improves Its Therapeutic Efficacy

Giulia Vindigni, Sofia Raniolo, Federico Iacovelli, Valeria Unida, Carmine Stolfi, Alessandro Desideri and Silvia Biocca

Table S1. Sequences of the oligos for the assembly of octahedral DNA nanocages. Unmodified oligonucleotides were HPLC purified and purchased from Integrated DNA Technologies. $OL6_{BIO}$ and $OL8_{APT}$ were purchased from LGC Biosearch Technologies. AS1411 was purchased from Sigma Aldrich. The 5′ of each oligo is phosphorylated. TTTTT represents a short non-pairing spacer inserted within the strands as a DNA junction at each vertex of the assembled 3D structure. $OL6_{BIO}$ has a biotin tetra-ethylen-glycol molecule (BtndT) at the T represented in red.

Oligo	Sequence (5′-3′)
OL1	GCCACCAGGTTTTTCGATGTCTAAGCTGACCGTTTTTGGACCGTGATTCCATGACTTTTTCTTAGAGTT
OL2	TGGCTACAGTTTTTCGGTCAGCTTAGACATCGTTTTTGAATCCTATGCTCGGACGTTTTTGGCTCACAT
OL3	TCACGGTCCTTTTTCTATCCGATCGAGGCATGTTTTTCATACTGAGAGCGTTCCGTTTTTGTCATGGAA
OL4	CAGATACGCTTTTTCATGCCTCGATCGGATAGTTTTTCTGTAGCCAATGTGAGCCTTTTTGTCGCAGTT
OL5	CTCAGTATGTTTTTCGGTTACGGTACAATGCCTTTTTCGCAAGACGTTAGTGTCCTTTTTCGGAACGCT
$OL6_{BIO}$	GGTGTATCGTTTTTGGCATTG**T**ACCGTAACCGTTTTTGCGTATCTGAACTGCGACTTTTTCCACCGAAT
OL7	CGTCTTGCGTTTTTGTATGACGCAGCACTTGCTTTTTCCTGGTGGCAACTCTAAGTTTTTGGACACTAA
OL8	ATAGGATTCTTTTTGCAAGTGCTGCGTCATACTTTTTCGATACACCATTCGGTGGTTTTTCGTCCGAGC
$OL8_{APT}$	ATAGGATTCTTTTTGCAAGTGCTGCGTCATACTTTTTCGATACACCATTCGGTGGTTTTTCGTCCGAGCTTTTTTT GGTGGTGGTGGTTGTGGTGGTGGTGG
AS1411	GGTGGTGGTGGTTGTGGTGGTGGTGG

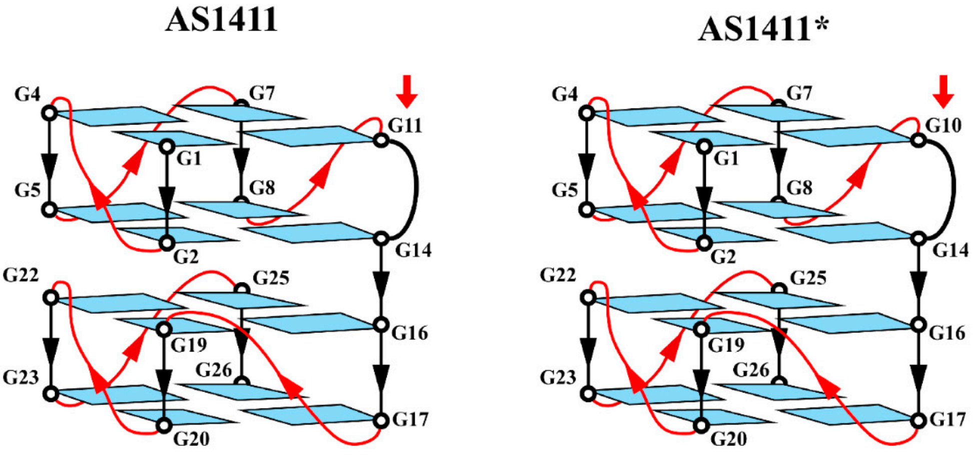

Figure S1. Schematic representation of G-quadruplex folding topology of AS1411 and AS1411* in K^+ solution. The red arrows indicate the guanines in position 11 and 10, responsible for the two distinct conformations assembly of the aptamer.

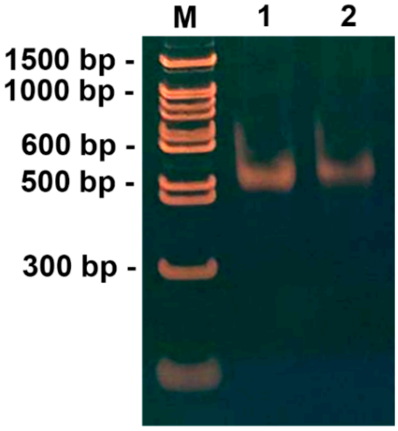

Figure S2. Gel electrophoresis analysis of pristine (NCs) and AS1411-functionalized nanocages (Apt-NCs). 100 ng of DNA nanocages were run in 5% polyacrylamide gel electrophoresis in TAEM buffer (40 mM Tris-acetic acid pH 7.0, 1 mM EDTA, 12.6 mM magnesium acetate) following standard procedures. Assembled NCs (lane 1) and Apt-NCs (lane 2) run as single band with a MW of approximately 530 bp. Notice that Apt-NCs has a slight difference in electrophoretic mobility due to the presence of AS1411 linked to the structure. The SharpMass 100 bp DNA ladder was purchased from Euroclone (Euroclone, Devon, UK).

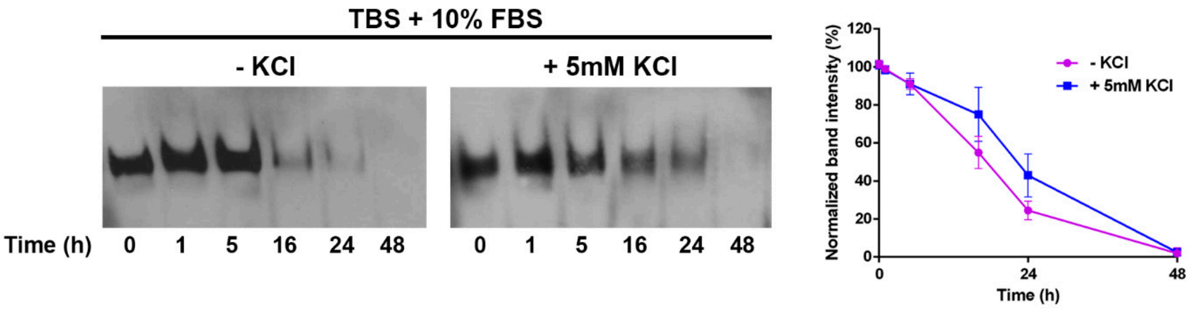

Figure S3. Stability of Apt-NCs in the absence or presence of K^+ ions.

Apt-NCs were incubated in TBS (Tris-HCl 50 mM, NaCl 150 mM, pH 7.8) supplemented with 10% FBS in the absence or in the presence of 5mM KCl for different time points at 37 °C, as indicated. Biotinylated Apt-NCs were detected with streptavidin-HRP. The densitometric analysis was performed by using ImageJ software. The relative intensity of each band was normalized to the intensity of the band corresponding to each time 0 and reported in the graph. The graph shows average ± SEM of three different experiments.

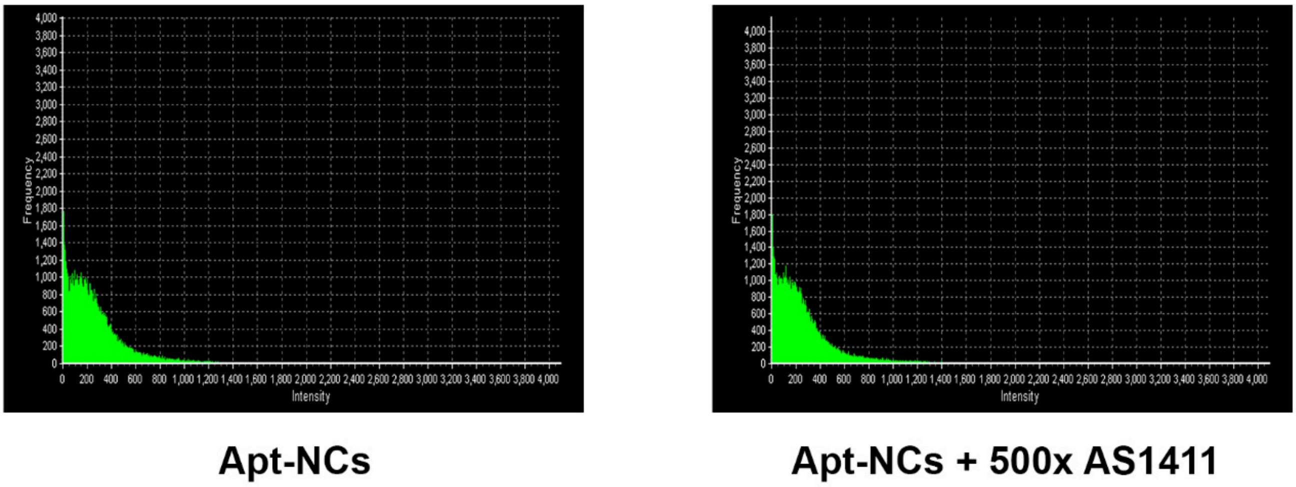

Figure S4. Intensity profiles of streptavidin-FITC signal.

HeLa cells were incubated with biotinylated Apt-NCs in the absence or in the presence of 500 times molar excess of free AS1411 for 1 h at 37 °C. Biotinylated Apt-NCs were visualized with streptavidin-FITC and analyzed by confocal microscopy. The green fluorescence signals of each condition tested were evaluated with Olympus FV1000 software and the fluorescence intensity profiles are reported in the graphs.

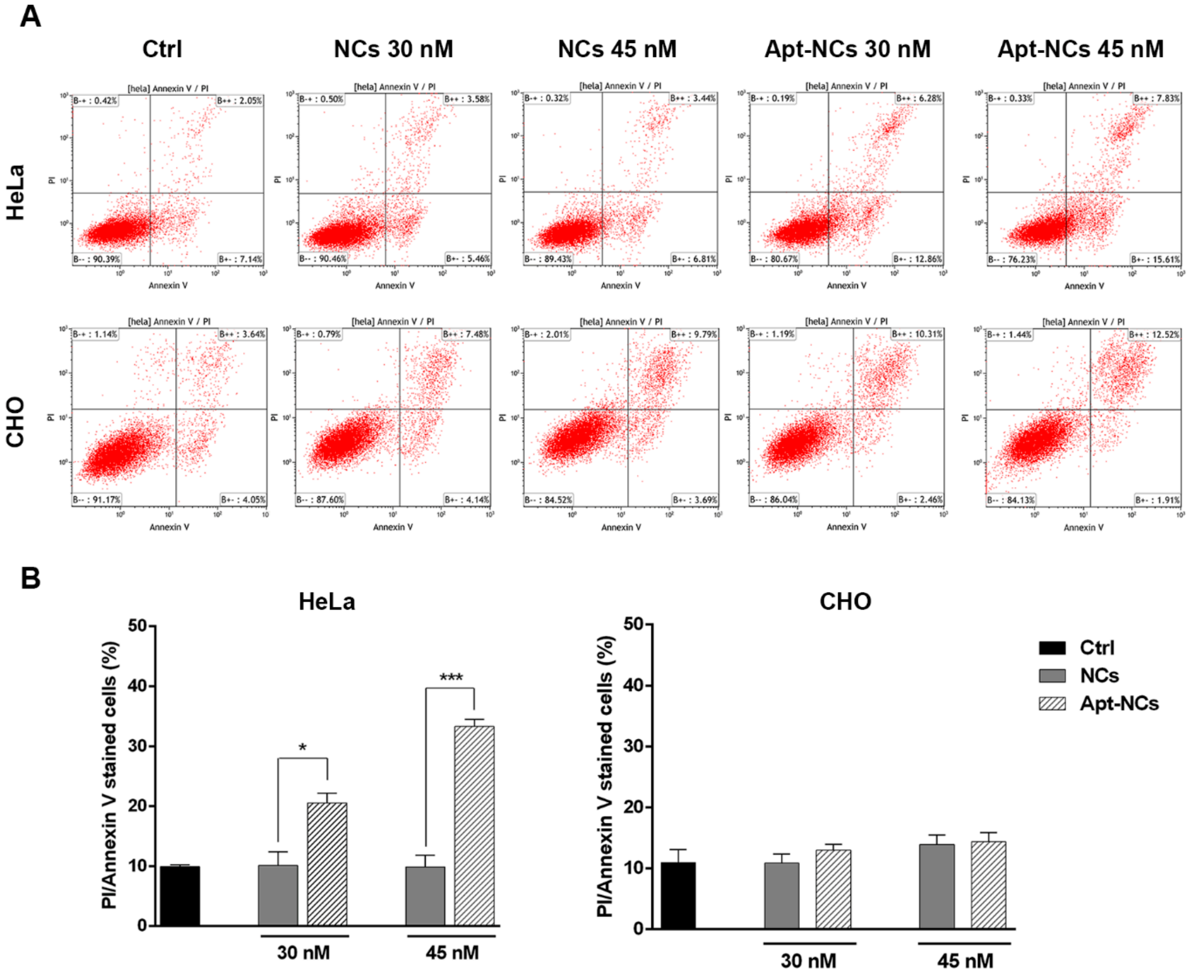

Figure S5. Annexin V-FITC/Propidium Iodide assay in HeLa and CHO cells.Cells were incubated for 24 h at 37 °C with different concentration of not-functionalized NCs and Apt-NCs, as indicated. After treatment, cells were harvested and then were double stained with Annexin V-FITC

and Propidium Iodide (PI) for flow cytofluorimetric analysis. Control cells (Ctrl) were incubated with culture media. For each condition tested, 10^4 cells were analyzed. (**A**) Representative flow cytometry plots. The fluorescence intensity of Annexin V/FITC is plotted on the x-axis, and PI is plotted on the y-axis. Cells were classified as healthy cells (Annexin V^-, PI^-), early apoptotic cells (Annexin V^+, PI^-), late apoptotic cells (Annexin V^+, PI^+), and damaged cells (Annexin V^-, PI^+). (**B**) Percentage of Annexin V-FITC/PI stained HeLa and CHO cells. Histograms show average values ± SEM of three independent experiments. Statistical significance: *$p < 0.05$ and ***$p < 0.001$ (Student's *t*-test).

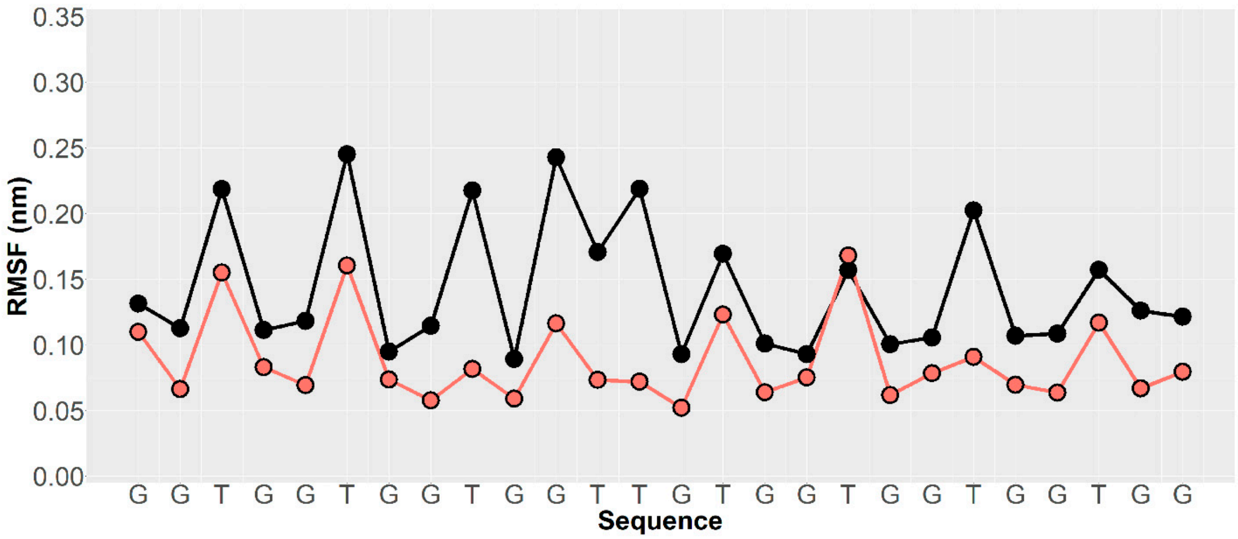

Figure S6. RMSF values calculated for the C2′ atoms of the AS1411* structures. Red and black filled circles indicate the values calculated for free and cage-linked AS1411*, respectively.

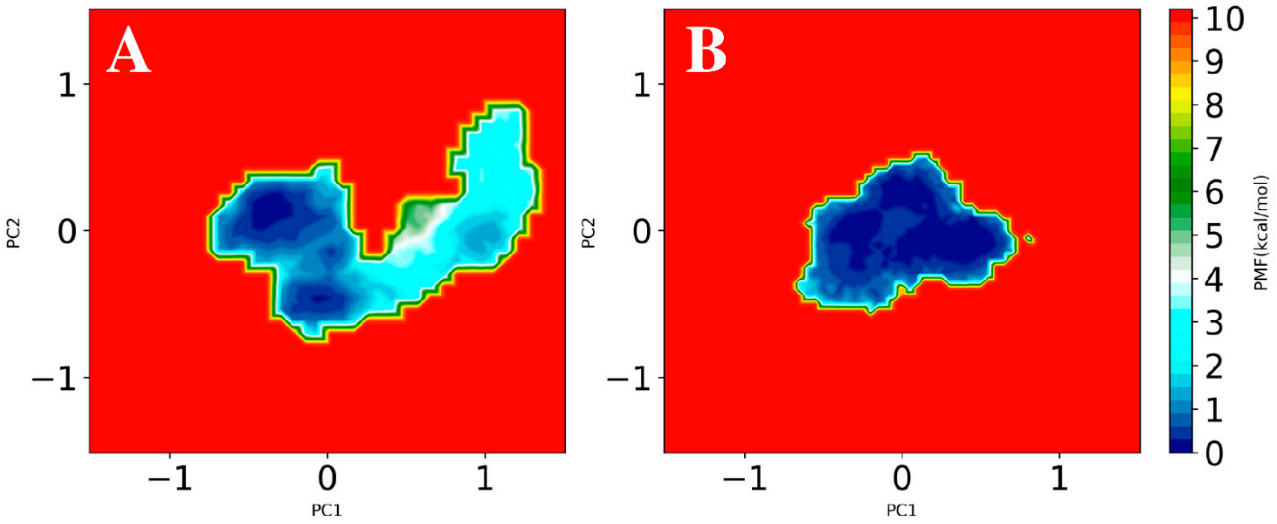

Figure S7. Free energy principal component projection of the isolated (**A**) and cage-linked (**B**) AS1411* aptamers, respectively.

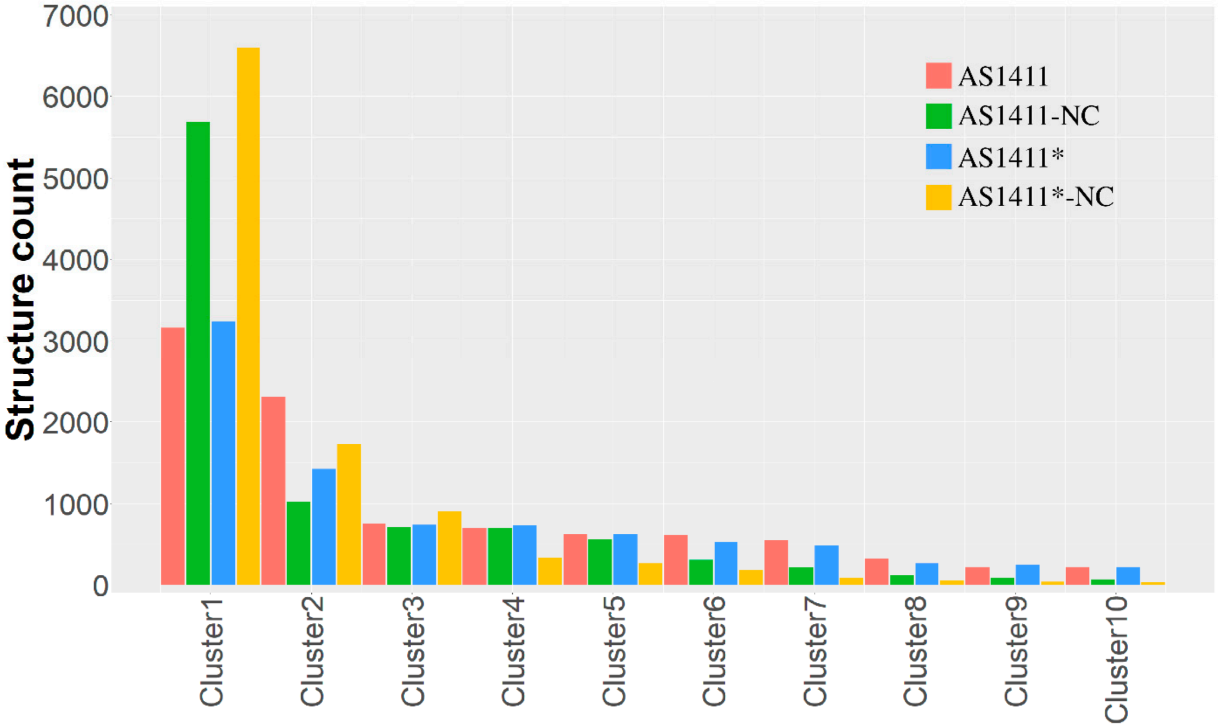

Figure S8. Bar chart representing the distribution of the clustered AS1411, AS1411-NC, AS1411* and AS1411*-NC conformations (red, green, blue and yellow bars, respectively) calculated on 10000 structures extracted from the trajectories. Only the first ten clusters are shown for convenience.
